# COVID-19 e Incertezas: Lições do Frontline para a Promoção da Decisão Compartilhada

**DOI:** 10.36660/abc.20200582

**Published:** 2020-08-19

**Authors:** José Augusto Soares Barreto-Filho, André Veiga, Luis Claudio Correia

**Affiliations:** 1 Universidade Federal de Sergipe Programa de Pós-Graduação em Ciências da Saúde Aracaju SE Brasil Universidade Federal de Sergipe - Programa de Pós-Graduação em Ciências da Saúde,Aracaju, SE - Brasil; 2 Universidade Federal de Sergipe Departamento de Medicina São Cristovão SE Brasil Universidade Federal de Sergipe - Departamento de Medicina,São Cristovão, SE - Brasil; 3 Hospital São Lucas Rede São Luiz D’Or Aracaju SE Brasil Emergência Cardiológica do Hospital São Lucas Rede São Luiz D’Or,Aracaju, SE - Brasil; 4 Escola Bahiana de Medicina e Saúde Pública Salvador BA Brasil Escola Bahiana de Medicina e Saúde Pública,Salvador, BA – Brasil

**Keywords:** COVID-19, Coronavirus, Pandemia, Medicina Baseada em Evidências, Bioética, Tomada e Decisão Clínica, Decisão Compartilhada, Uso Off Label

A COVID-19 já é a maior e mais mortal epidemia dos últimos cem anos. Profissionais da saúde, na linha de tiro, são cobrados, diuturnamente, a dar respostas e tomar decisões que impactam diretamente a vida dos infectados e os cientistas convocados para a tarefa hercúlea de, em tempo recorde, oferecer “remédios eficazes” para uma virose recém descoberta, e com um potencial mortal devastador. Com uma avalanche de informações nunca vista, o debate de como tratar os pacientes com a COVID-19 transcendeu a arena técnica e se tornou também ideológico e político.

A ciência se lastreia em fatos. E o fato é que não temos disponível tratamento etiológico com eficácia e segurança comprovadas para combater o SARS-CoV-2. Por enquanto, apenas promessas no *pipeline* . Para exemplificar, o caso mais emblemático da falta de racionalidade no pensamento científico é a polêmica acerca da cloroquina/hidroxicloroquina (CQ/HCQ) no tratamento da COVID-19. A CQ/HCQ é uma droga largamente utilizada, com sucesso, em portadores de malária e de lúpus eritematoso sistêmico. Contra a COVID-19, a droga impede a replicação do SARS-CoV-2 *in vitro* e modula a cascata inflamatória desencadeada pelo vírus.^1^ Dados *in vitro* trazem plausibilidade biológica, porém plausibilidade não é o mesmo que probabilidade da hipótese ser verdadeira. A CQ/HCQ foi alçada à categoria de bala mágica por uma publicação francesa^2^ cuja metodologia apresenta alto risco de viés e erro aleatório, não podendo ser definida como “evidência científica”. Entretanto, tal publicação foi superestimada, de forma crente ou ideológica, pelos menos fiéis aos preceitos e à liturgia da ciência. Contaminados por essa falácia e sentindo-se na obrigação de solucionar magicamente a pandemia, até presidentes se prestaram ao papel de propagandistas de fármacos, ajudando a virilizar uma pseudociência e ampliar a problemática de informações falsas.

Mesmo na comunidade médico-científica, o debate também se tornou pouco racional e ideologizado. Uma das alegações dos entusiastas da CQ/HCQ era que, em um cenário de guerra, dever-se-iam utilizar as armas que estão disponíveis, mesmo sem a comprovação definitiva de eficácia e/ou segurança clínica. Contrariando a máxima *“Primum Non Nocere”* , fazer mal seria perdoado, a inércia jamais. Em outro polo, alguns embarcaram no debate maniqueísta ao valorizar, também de forma irracional, os estudos observacionais para argumentar prova de ineficácia. A ferrenha defesa do uso da CQ/HCQ seduz, pois existem efeitos fisiopatológicos plausíveis, verificados em laboratório, para se acreditar que a droga é eficaz. Porém, sua eficácia clínica não foi comprovada em nenhum modelo patológico de infecção viral aguda em humanos e muito menos na COVID-19.^3^

O efeito finalístico de uma droga depende da resultante dos seus efeitos positivos e negativos. E tal resultante pode desencadear um efeito finalístico neutro (tratamento fútil), positivo (tratamento eficaz) ou negativo (tratamento maléfico). Antes do escrutínio científico rigoroso, não há como se prever. A função do ensaio clínico randomizado é provar, com precisão probabilística pela estatística, que a droga A causa melhora do paciente portador de uma doença B e não causa efeitos colaterais impeditivos à sua prescrição.

Em um ecossistema científico organizado, conhecimentos prévios embasam os estudos futuros através de uma probabilidade condicional. Hipóteses improváveis, ainda não confirmadas, quando adotadas como política de saúde, ocasionam gastos desnecessários de recursos humanos e econômicos, geram falsas esperanças no inconsciente coletivo e, eventualmente, até significativo malefício.

Para o médico treinado a responder de forma proativa, essa incerteza, em um cenário de comoção coletiva, pode ser extremamente desconfortante e, no desejo inconsciente de resolver o seu drama interno da impotência médica, ser traído por vieses cognitivos. Sendo a premissa contemporânea do nosso ofício crer em uma Medicina alicerçada na boa ciência, precisamos pausar nossas mentes agitadas pelo *tsunami* da pandemia para uma reflexão mais lúcida, lógica e iluminada pelo nosso credo. A história da ciência biomédica já devia ter nos ensinado, enquanto comunidade científica, que o desvio dos caminhos da ciência formal pode nos levar a um “curto caminho longo”. A busca por um atalho, no calor do desespero, poderá até contribuir com mortes que poderiam ser evitáveis, caso o potencial tóxico da CQ/HCQ, neste cenário, venha a ser verificado por ensaios clínicos randomizados.

Ser dogmático, promovendo a prescrição de drogas antes de testes de fase III, deve ser considerado heresia científica contemporânea. Cuidado não embasado em evidências não necessariamente representa um bom cuidado. A suposta “inércia” de não se prescrever uma terapia por carência de lastro na evidência é, na maioria das vezes, uma boa prática médica. Em tese, a probabilidade pré-teste de uma droga A, nunca testada em um determinado cenário, ser eficaz é muito remota. Daí a normatização de se usar o ensaio clínico experimental como palavra final. Não é incomum hipóteses lastreadas por suporte mecanístico ou estudos observacionais não serem confirmadas por ensaios randomizados.

Cláusula pétrea, na ciência, o ônus da prova encontra-se na comprovação da eficácia e não na comprovação da ineficácia e, assim, partimos da premissa inicial do pensamento científico, a hipótese nula deve ser formalmente rejeitada para a comprovação do fenômeno. Apenas o argumento de uma terapia ser segura não justifica o uso de uma droga ineficaz. A comprovação do benefício é condição essencial para se cotejar os resultados positivos com os eventuais riscos de uma determinada droga. No caso da CQ/HCQ, temos a seguinte situação: a maioria dos estudos observacionais aceitáveis não comprovaram o benefício da droga e sua segurança ainda não foi definida.

E qual seria o norte para a tomada de decisão com tantas incertezas, pressão pandêmica e ausência de evidências? Antes, é importante destacarmos que a falta de evidência de efeito não significa evidência de nenhum efeito. Negar, categoricamente, um potencial benefício não parece também ser o melhor caminho. É duvidoso se CQ/HCQ tem uma probabilidade a priori que justifique grandes esforços científicos. Mas, mesmos para casos em que essa probabilidade seja razoável, a primeira opção seria em se comprometer com a tarefa de direcionar pacientes para serem alocados em ensaios clínicos. Um esforço coletivo, solidário e articulado poderia encurtar os tempos das incertezas.

Não sendo possível, é compreensível, em uma situação de “guerra”, a proposta de uso *off-label* de fármacos, situação quando uma determinada droga, já devidamente registrada e aprovada em um cenário A, é liberada para um cenário B, sem estudos específicos; ou até o seu uso por compaixão (ou compassivo), quando uma droga, ainda experimental e sem qualquer registro junto a uma agência regulatória, é prescrita por falta de uma opção melhor e crença de que possa funcionar. Vale salientar que o uso compassivo é mais um ato de piedade que uma aposta no sucesso terapêutico.

No calor e desespero atual, estamos vivenciando um pandemônio caracterizado por uma proliferação, sem precedentes, de informações de péssima qualidade e grande variabilidade na prática prescritiva observada na linha de frente. Entretanto, as diretrizes e os editoriais publicados nas revistas científicas mais conceituadas são categóricos em afirmar que ainda não temos terapias etiológicas efetivas cientificamente comprovadas em reduzir a mortalidade da COVID-19.^1^ O tratamento de pneumonia viral continua a ser, basicamente, o de suporte e de intervenção nas diversas complicações clínicas que poderão surgir em uma minoria de pacientes. Reinventar o conhecimento, sobejamente alicerçado, e abandonar a liturgia do que reza a ciência clínica moderna parece ser um grande retrocesso à Idade das Trevas.

E como tomar tal decisão, quando a incerteza é a regra? Assumir uma postura autoritária ou paternalista não é a rota mais prudente. A atual situação na qual nos encontramos talvez seja uma oportunidade única de por em prática o princípio da autonomia do paciente, iluminando a tomada de decisão médica.

Historicamente, os pacientes sempre confiaram a tomada de decisões aos médicos. No entanto, durante as últimas décadas, pacientes tem sido incentivados a se tornarem ativos e participativos nas suas decisões sobre saúde. O documento “Crossing the Quality Chasm”, chancelado pelo Instituto de Medicina Americano, incentiva que uma voz ativa seja dada ao paciente em tudo que impactará sobre a sua vida. Operacionalmente, isso inclui informação transparente das expectativas e incertezas antes da tomada de decisão compartilhada. Embora entendamos a complexidade de se implantar um processo de decisão compartilhada na atual situação, a prescrição compulsória e indiscriminada de fármacos, sem eficácia e/ou segurança comprovadas neste cenário, não se afina com os valores atualmente preconizados. Interessante é que o princípio da autonomia do paciente se constitui em atributo que alicerça as bases do SUS, desde a sua fundação, e se alinha aos preceitos contemporâneos da Bioética.

A autonomia corresponde à capacidade das pessoas decidirem alinhadas aos seus próprios valores. A base da autonomia reside no respeito aos direitos fundamentais do indivíduo, considerando-o um ser biopsicossocial e espiritual, dotado de capacidade para tomar suas próprias decisões. Em momento de pandemia, quando a incerteza se torna ainda mais evidente, o resgate desse princípio fundamental, dando voz aos pacientes na mesa de decisão, pode ser a ponte para que o binômio médico-paciente escolha o melhor caminho customizado às expectativas do maior interessado pelos bons resultados, o paciente. Assumir integralmente o controle de todas as decisões médicas, nos auto-enganando com uma certeza inexistente, pode ser sinal de imaturidade. É urgente transpormos o modelo hipocrático, no qual o médico deveria aplicar “ *os regimes para o bem dos doentes, segundo seu saber e razão* ...”, sem conceder um lugar à autonomia destes, para um modelo de assistência centrada no paciente e compartilhado.

O momento atual exige um profissional atualizado, seguro, disponível para dialogar e convencer, de forma transparente, quais são as evidências factuais para uma tomada de decisão compartilhada. Separar o que é evidência científica dentro de tanta pseudociência clínica será tarefa cardinal. Ciência não se sustenta na fé, na crença e nem em opinião de autoridade. Pelo contrário, a dúvida e a incerteza são as principais motivações para se fazer a ciência avançar. É imprescindível perceber que as consequências das nossas decisões não são e não podem ser compartilhadas. Portanto, a prática médica para o enfrentamento da COVID-19 exige humildade em reconhecer as fronteiras do conhecimento científico atual. Compartilhar, com transparência, as incertezas e dúvidas, com os pacientes, poderá iluminar a tarefa, por demais pesada, de tomar decisões nesse atual cenário de muita escuridão. Essa parece ser uma grande oportunidade de aprendermos hoje e levarmos para o amanhã lições importantes que pavimentarão a utopia de uma “medicina que serve os doentes”.
