# Supplementary material for: COVID-19 e Incertezas: Lições do Frontline para a Promoção da Decisão Compartilhada
Source: Arq Bras Cardiol. 2020 Aug 19;115(2):149–51. [Article in Portuguese] doi: 10.36660/abc.20200582 (PMC8384286; doi:10.36660/abc.20200582)
Supplement: Translation

# Translation: COVID-19 and Uncertainty: Lessons from the Frontline for Promoting Shared Decision Making

**Authors:** José Augusto Soares Barreto-Filho, André Veiga, Luis Claudio Correia

**Affiliations:** 1 Brazil Universidade Federal de Sergipe - Programa de Pós-Graduação em Ciências da Saúde, Aracaju, SE - Brazil; 2 Brazil Universidade Federal de Sergipe - Departamento de Medicina, São Cristovão, SE - Brazil; 3 Brazil Emergência Cardiológica do Hospital São Lucas Rede São Luiz D’Or, Aracaju, SE - Brazil; 4 Brazil Escola Bahiana de Medicina e Saúde Pública, Salvador, BA – Brazil

**Keywords:** COVID-19, Coronavirus, Pandemics, Evidence Based Medicine, Bioethics, Clinical Decision Making, Decision Making,Shared, Off Label Use

COVID-19 has already become the largest and deadliest epidemic of the past hundred years. On a daily basis, healthcare professionals in the frontline are called upon to give answers and make decisions that directly affect the lives of infected patients, and scientists are summoned to the Herculean task of providing “effective medications” in record time for a recently discovered virus with devastating potential mortality. With a hitherto unseen avalanche of information, the debate on how to treat patients with COVID-19 has gone beyond the limits of the technical arena, taking on ideological and political aspects as well.

Science is based on facts. The fact is that we do not currently have an etiological treatment with proven efficacy and safety to combat SARS-CoV-2. At the moment, there are only promises in the pipeline. To exemplify, the most emblematic case of lack of rationality and scientific thinking is the polemic regarding chloroquine/hydroxychloroquine (CQ/HCQ) for treatment of COVID-19. CQ/HCQ is a drug that has been widely and successfully used in patients with malaria and systemic lupus erythematosus. Against COVID-19, the drug inhibits replication of SARS-CoV-2 *in vitro* , and it modulates the inflammatory cascade triggered by the virus.^1^ *In vitro* data demonstrate biological plausibility, but plausibility does not mean likelihood that a hypothesis is true. CQ/HCQ was, nevertheless, promoted to the category of “magic bullet” by a publication from France,^2^ whose methodology was characterized by high risk of bias and random error, meaning that it could not be defined as “scientific evidence.” This notwithstanding, the publication was overestimated, in an ideological manner, by the individuals who were least faithful to the precepts of the liturgy of science. Contaminated by this fallacy, feeling obligated to solve the pandemic magically, even presidents took on the role of drug advertisers, thus helping to viralize pseudoscience and amplify the false information problem.

Even within the medical scientific community, the debate has also become ideological and scarcely rational. One of the claims of CQ/HCQ enthusiasts was that, in a scenario of war, it is necessary to use whatever weapons are available, even without definitive proof of their efficacy and/or clinical safety. Going against the maxim *“Primum Non Nocere,* ” they deemed that it was forgivable to do harm, but not to remain inert. On the other side, some embarked on a Manichean debate by emphasizing, also irrationally, observational studies in order to argue that there was proof that it was ineffective. The unwavering defense of CQ/HCQ is seductive, given that there are plausible physiopathological effects, which have been verified in the laboratory, suggesting that the drug is effective. Nevertheless, its clinical efficacy has not been proven in any pathological model of acute viral infection in humans, much less with respect to COVID-19.^3^

The final effect of a drug depends on the result of its positive and negative effects. The results may trigger a final effect that is neutral (futile treatment), positive (effective treatment), or negative (harmful treatment). Before rigorous scientific scrutiny has been applied, it is not possible to predict them. The function of a randomized clinical trial is to prove, with probabilistic accuracy, using statistics, that drug A causes improvement in patients with disease B and that it does not have side effects which would contraindicate prescription.

In an organized scientific ecosystem, prior knowledge provides a basis for future studies through conditional probability. Unlikely hypotheses, which have not been confirmed, when adopted as health policies, lead to unnecessary expenditure of human and economic resources, and they generate false hope in the collective unconscious in addition to, eventually, significant harm.

For physicians who are trained to respond proactively, this uncertainty, in scenarios of collective turmoil, may be extremely disconcerting and, driven by the unconscious desire to resolve their internal conflicts related to medical impotence, they may be betrayed by cognitive biases. Given that the contemporary premise of our vocation is to believe in Medicine based on good science, we need to offer a moment’s rest to our minds, which have been troubled by pandemic tsunami so that we may reflect more lucidly, logically, and in a manner enlightened by our creed. The history of biomedical science should have already taught us, as a scientific community, that straying from the paths of formal science can lead us down a “long shortcut.” The search for a shortcut, in the heat of despair, can even contribute to deaths that could have been avoided, in the event that the toxic potential of CQ/HCQ, in this scenario, is proven by randomized clinical trials.

From a dogmatic point of view, promoting prescription of drugs before phase III tests should be considered contemporary scientific heresy. Care that is not based on evidence does not necessarily represent good care. The alleged “inertia” of not prescribing a therapy in the absence of supporting evidence is, in most cases, good medical practice. In theory, the pre-test probability of a drug that has never been tested in a determined scenario being effective therein is very remote. Hence the standard of considering experimental clinical trials as the final word. It is not uncommon for hypotheses that are sustained by mechanistic support or observational studies not to be confirmed in randomized trials.

A core principle in science is that the burden of proof lies in demonstrating efficacy, rather than inefficacy; for this reason, we begin with the initial premise of scientific thinking, namely, the null hypothesis must be formally rejected in order to prove the phenomenon. The argument that a therapy is safe alone does not justify implementing an ineffective drug. Proof that a benefit exists is a fundamental condition in order to compare a given drug’s positive results with its eventual risks. In the case of CQ/HCQ, we have seen the following situation: The majority of acceptable observational studies have not proven the benefit of the drug.

What, then, will serve as a compass for decision making with so much uncertainty and pandemic pressure, in the absence of evidence? First, it is important to underscore that lack of evidence regarding effect does not mean evidence that there is no effect. To deny a potential benefit categorically does not seem to be the best way, either. It is doubtful whether CQ/HCQ has a priori probability that justifies major scientific effort. Nonetheless, even in cases where there is a reasonable likelihood, the first option would be to commit to the task of selecting patients for allocation into clinical trials. Collective, solidary, and articulated efforts could shorten the duration of this uncertainty.

When this is not possible, it is understandable, in situations of “war,” to propose off-label use of medications, when a specific drug that has already been properly registered and approved for scenario A is permitted for scenario B without specific studies, or even compassionate use, when a drug that is still experimental and that has not been registered by any regulatory agency is prescribed for lack of a better option in the belief that it might work. It is necessary to underscore that compassionate use is more an act of mercy than a bet on therapeutic success.

In the heat of this desperate moment, we are experiencing a pandemonium characterized by the unprecedented proliferation of information of the worst quality, with great variability in the prescription practice observed in the frontlines. In the meanwhile, the guidelines and editorials published in the most prestigious scientific journals have categorically stated that we do not yet have effective etiological therapies that are scientifically proven to reduce the mortality of COVID-19.^1^ The treatment of viral pneumonia continues, essentially, to be that of support and intervention in the diverse clinical complications that may arise in a minority of patients. To reinvent knowledge, which is substantially well founded, and to abandon the liturgy of modern clinical science appears to be a great retrogression to the Dark Ages.

How, then, shall we make decisions, when uncertainty is the rule? To take an authoritarian or paternalistic stance would not be the wisest path. The current situation in which we find ourselves may perhaps be a unique opportunity to put the principle of patient autonomy into practice, thus enlightening medical decision making.

Historically, patients would entrust decision making to physicians. During the last decades, however, patients have been encouraged to take an active role and to participate in decisions about their health. The *Crossing the Quality Chasm* report, published by the American Institute of Medicine, argues that an active voice should be given to patients in respect to all that will have an impact on their lives. Operationally speaking, this includes transparent information regarding expectations and uncertainties, before shared decision making. Although we understand the complexity of implementing a shared decision-making process during the current situation, the compulsory and indiscriminate prescription of drugs that have no proven efficacy and/or safety for this scenario does not corroborate the values currently put forth. It is noteworthy that the principle of patient autonomy is an attribute that underpins the basis of the Brazilian Unified Health System, since its foundation, and it is in line with precepts of contemporary Bioethics.

Autonomy corresponds to people’s capability to decide in accordance with their own values. The basis of autonomy resides in respect for individuals’ fundamental rights, considering them as biopsychosocial and spiritual beings endowed with the ability to make their own decisions. During a pandemic, when uncertainty becomes even more evident, the return to this fundamental principle of giving patients a voice in the decision table may serve as a bridge whereby the physician-patient binomial will be able to choose the best path, customized to the expectations of the person who is most interested in positive outcomes, namely, the patient. To take full control of all medical decisions and to deceive ourselves with certainties that do not exist can be a sign of immaturity. It is urgent that we transcend the Hippocratic model, wherein physicians are to apply “regimens for the good of patients, according to their knowledge and reason,” leaving no room for their autonomy, to a shared, patient-centered model of care.

The current moment calls for professionals who are up-to-date, confident, and open to transparent dialogue on factual evidence in favor of shared decision making. Separating what is scientific evidence, in the midst of so much clinical pseudoscience, will be the cardinal task. Science is not based on faith, belief, opinion, or authority. On the contrary, doubt and uncertainty are the main reasons behind advances in science. It is indispensable to recognize that the consequences of our decisions are not and cannot be shared. Therefore, medical practices for dealing with COVID-19 require humility in that we recognize the boundaries of current scientific knowledge. Transparent sharing of uncertainties and doubts with patients will make it possible to shine a light on the otherwise excessively cumbersome task of making decisions in this scenario, in which there is still too much darkness. This seems to be a significant opportunity to learn today and to bring important lessons to tomorrow in order to pave the way toward the utopia of “medicine that serves patients”.^7^
